# An Integrated Approach to Falls Prevention: A Model for Linking Clinical and Community Interventions through the Massachusetts Prevention and Wellness Trust Fund

**DOI:** 10.3389/fpubh.2017.00038

**Published:** 2017-03-06

**Authors:** Laura J. Coe, Julie Ann St. John, Santhi Hariprasad, Kalpana N. Shankar, Patricia A. MacCulloch, Amy L. Bettano, Jean Zotter

**Affiliations:** ^1^Prevention and Wellness Trust Fund, Bureau of Community Health and Prevention, Massachusetts Department of Public Health, Boston, MA, USA; ^2^Graduate School of Biomedical Sciences, Texas Tech University Health Sciences Center, Abilene, TX, USA; ^3^JSI Research & Training Institute, Inc., Boston, MA, USA; ^4^Emergency Medicine, Boston Medical Center, Boston University, USA; ^5^School of Nursing, University of Massachusetts Lowell, Lowell, MA, USA

**Keywords:** older adult fall prevention, clinical and community linkage, Massachusetts Prevention and Wellness Trust Fund, implementing Stopping Elderly Accidents, Deaths and Injuries, community-based fall prevention

## Abstract

Older adult falls continue to be a public health priority across the United States—Massachusetts (MA) being no exception. The MA Prevention and Wellness Trust Fund (PWTF) program within the MA Department of Public Health aims to reduce the physical and economic burdens of chronic health conditions by linking evidence-based clinical care with community intervention programs. The PWTF partnerships that focused on older adult falls prevention integrated the Centers for Disease Control and Prevention’s *Stopping Elderly Accidents, Death and Injuries* toolkit into clinical settings. Partnerships also offer referrals for home safety assessments, Tai Chi, and Matter of Balance programs. This paper describes the PWTF program implementation process involving 49 MA organizations, while highlighting the successes achieved and lessons learned. With the unprecedented expansion of the U.S. Medicare beneficiary population, and the escalating incidence of falls, widespread adoption of effective prevention strategies will become increasingly important for both public health and for controlling healthcare costs. The lessons learned from this PWTF initiative offer insights and recommendations for future falls prevention program development and implementation.

## Background

### Burden of Older Adult Falls

One-third of adults aged 65 years and older experience a fall each year and the risk increases proportionally with age (1). Non-fatal falls continue to pose a significant social and economic burden on individuals and the healthcare system, as fall-related deaths among this population have more than doubled over the last decade (2). In 2013, non-fatal falls led to 2.8 million emergency department (ED) visits with nearly one-third requiring hospital admission, incurring $34 billion in associated healthcare costs (2, 3).

Many falls can be prevented through the widespread adoption of evidence-based clinical practice guidelines, the integration of a home safety assessment, and the implementation of community-based falls prevention interventions (4). Unfortunately, despite the evidence of their effectiveness, these interventions have not found widespread use in clinical or community practice (1, 3, 5). Older adults who have experienced a fall and receive emergency medical evaluations seldom receive a clinical falls risk assessment at the time of treatment, and few are referred for falls prevention interventions. The creation of a coordinated and comprehensive system of care for falls prevention is possible and can be achieved with a paradigm shift for clinicians, new collaborations with community stakeholders, and a payment model to support these interventions.

In the current national effort to both transform healthcare through Accountable Care Organizations (ACOs) and restructure payments based on value instead of volume, there is an unprecedented opportunity and incentive to integrate evidence-based community falls prevention programs with clinical care.

### The Prevention and Wellness Trust Fund (PWTF)

A state law enacted in 2012, “An Act Improving the Quality of Health Care and Reducing Costs through Increased Transparency, Efficiency and Innovation,” Chapter 224 of the Acts of 2012 (6) established the PWTF. The law aims to control healthcare cost growth through a number of mechanisms including the creation of the PWTF to invest in wellness and prevention. PWTF focuses on reducing rates of the most prevalent and preventable health conditions in the state by using cost-effective, evidence-based interventions and linking patients between clinical and community domains.

The PWTF program, administered by the Massachusetts Department of Public Health (MDPH), invested $42.75 million over a 4-year period in nine community partnerships across the state to reduce the burden of pediatric asthma, hypertension, tobacco, and older adult falls. The total population served by the nine partnerships comprises roughly 987,400 residents (approximately 15% of the state’s population).

**Figure 1:**
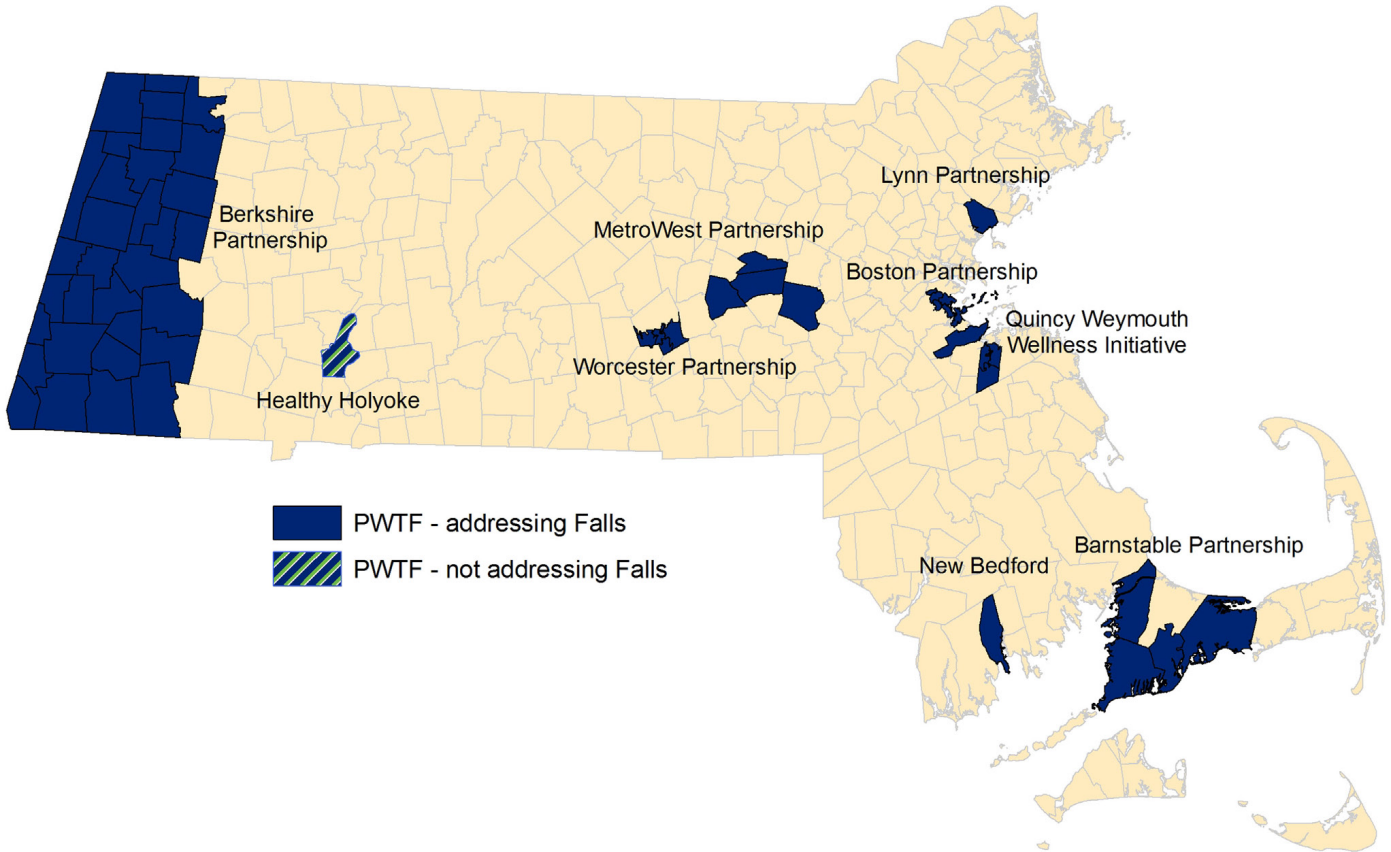
**Prevention and Wellness Trust Fund (PWTF) partnership map**.

Prevention and Wellness Trust Fund communities are distributed across the Commonwealth. They consist of both rural and urban regions, including some of the poorest, most racially and ethnically diverse populations in the state. Populations within these communities have higher percentages of non-English speakers and lower levels of primary education as compared to the rest of Massachusetts and the U.S. (refer to Table 1 and Figure 1). Each partnership includes clinical sites, community-based organizations, and municipalities.

**Table 1:** **Demographics**.^a^

	Prevention and Wellness Trust Fund communities (%)	Massachusetts (%)	United States (%)
**Race**			
White, NH	50.3	80.4	72.4
Black/African-American, NH	15.2	6.6	12.6
American Indian/Alaskan Native, NH	0.3	0.3	0.9
Asian, NH	5.8	5.3	4.8
Hawaiian Native/Pacific Islander, NH	0.3	0.0	0.2
Hispanic/Latino (any race)	21.8	9.6	16.3
**Gender**			
Total population—male	48.2	48.4	49.2
Total population—female	51.8	51.6	50.8
≥65 years—male	41.1	41.7	43.6
≥65 years—female	58.9	58.3	56.4
**Aged ≥65 years**	14.8	14.4	13.7
**Persons with incomes below Federal Poverty Level (individuals ≥65 years)**	12.0	9.1	9.4
**Education (individuals ≥65 years)**			
High school not completed	22.0	18.4	20.0
High school degree or higher	78.0	81.6	80.0
**Speak a language other than English at home (individuals ≥18 years)**	21.0	16.4	14.4

*Sources: ^a^American Community Survey and US Census Data, US Census Bureau, 2008–2014 data. Prepared by the Massachusetts Department of Public Health*.

*NH, Non-Hispanic*.

### Innovative Approaches to Linking Clients with Preventative Community Programs

Recognizing individuals spend a majority of their time in their community (e.g., at home, in the neighborhood, at work, and in school), the PWTF model extends care from the clinical setting to the community by establishing communication and referral mechanisms between clinics and community organizations offering prevention programs. Connecting the two sectors optimizes prevention opportunities, reduces costs and fosters a shared responsibility for improving the safety and wellness of their population. Not only is this clinical-to-community collaboration model aligned with the Triple Aim (7) but it also charts a path toward future payment reform strategies that incentivize wellness and prevention.

Prevention and Wellness Trust Fund partnerships utilize several linkage strategies, including targeted use of trained Community Health Workers (CHWs); an innovative electronic referral system “e-Referral” that is embedded in the electronic medical record (EMR); and traditional methods such as secure fax. These linkage methods are bi-directional and the referring primary care clinicians are able to offer and receive feedback on patient participation and progress in the community intervention.

Community Health Workers employed by both PWTF clinical and community sites play a critical role not only in encouraging individuals to enroll in PWTF interventions, but in assisting clients in identifying and overcoming potential barriers to initiation, participation and completion of the interventions. CHWs typically live in or near the community they serve and often share the language and cultural background of the individuals they serve.

### PWTF Interventions for Falls Prevention Methods

**Figure 2:**
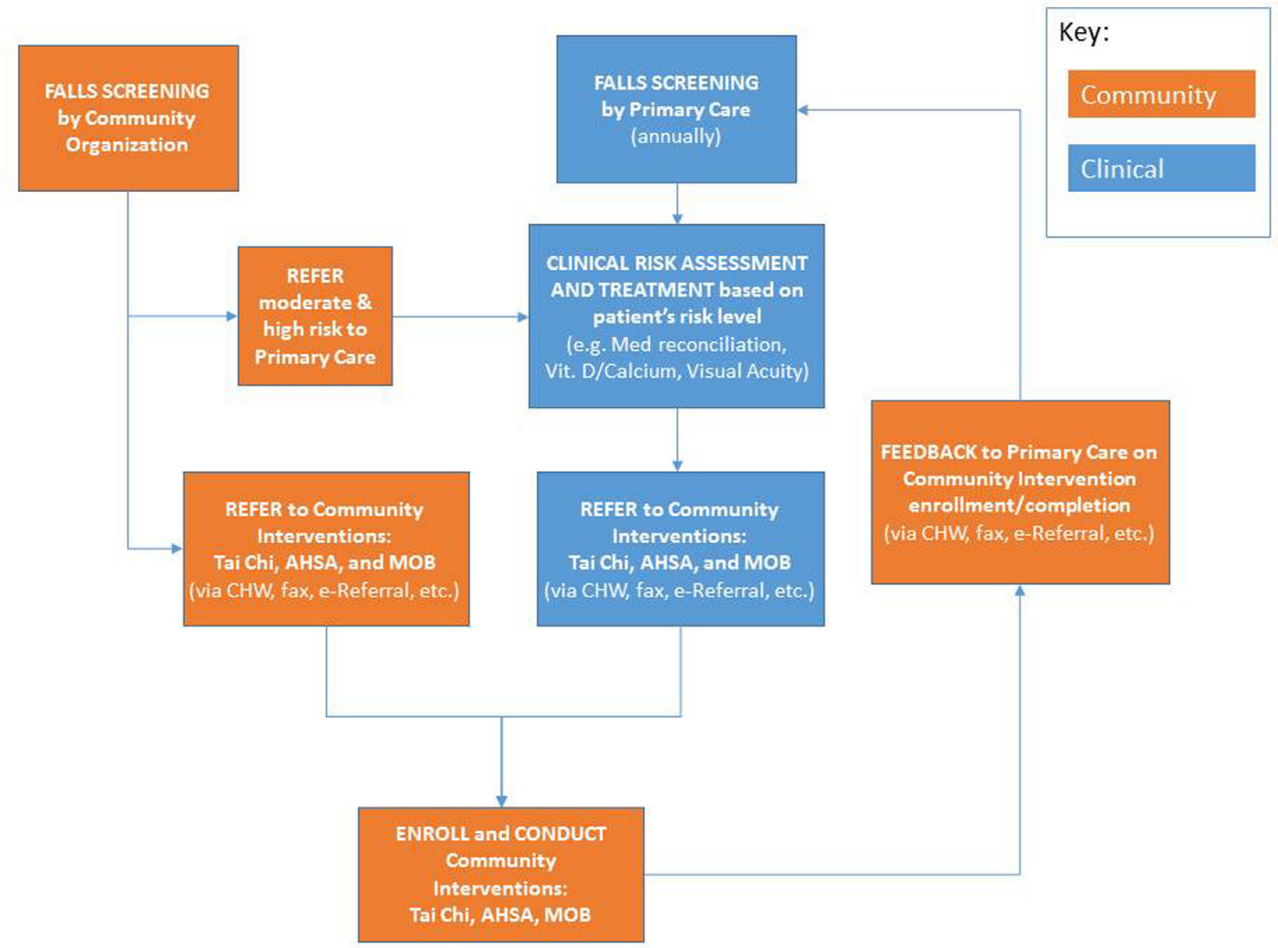
**PWTF fall prevention workflow**.

Eight of the nine PWTF partnerships selected falls as one of their priority conditions and are implementing evidence-based or evidence-informed interventions. The PWTF model for integrated falls prevention includes screening, clinical assessment and referral to community-based prevention interventions for individuals aged 65 years and older based on the CDC’s Stopping Elderly Accidents, Deaths and Injuries (STEADI) toolkit and algorithm (8) (refer to Figure 2). The community-based interventions, described in more detail below, are Tai Chi, Matter of Balance (MoB), and Assisted Home Safety Assessment and Modification (AHSA). These interventions complement each other and individuals at-risk for falls can benefit by participating in more than one of the interventions.

(1) *Stopping Elderly Accidents, Deaths and Injuries Toolkit*: the CDC developed the STEADI toolkit for primary care providers (PCPs), physical therapists (PTs), and other professionals who serve older adults (8). The toolkit includes the Algorithm for the Fall Risk Assessment & Intervention that is based on the American Geriatrics Society/British Geriatrics Society Clinical Practice Guideline for the Prevention of Falls in Older Persons (9, 10). The algorithm guides the provider through a standardized process for annual and/or acute falls risk screening, multifactorial assessment and appropriate referrals for both clinical issues and community-based falls prevention interventions. Each partnership tailored this algorithm to complement its own workflow to optimize the referral pattern. Multifactorial falls risk assessments, like STEADI, are associated with a reduction in annual falls among people who have fallen in the prior year (4).
(2) *Tai Chi*: Tai Chi is a traditional martial art that involves slow, flowing movements, and deep breathing. Tai Chi is highly effective at reducing the risk of falls in community-dwelling older adults (11). It promotes relaxation and improves muscle strength, stability, gait, posture, and coordination (11). Trained instructors deliver the program in 1-h sessions twice per week for 24 weeks. Each session consists of warm-up exercises, core practices or forms, and brief cool-down exercises. In particular, the Tai Chi: Moving for Better Balance program has a net benefit of $529.86 per participant and a return on investment (ROI) of 509% for every dollar invested based on the reductions in the direct medical costs of falls (12).
(3) *Matter of Balance*: MoB is an 8-week structured group intervention focusing on practical strategies to reduce the fear of falling and increase activity levels. MoB includes eight 2-h sessions for a small group led by a trained facilitator. MoB workshops include: group discussions; mutual problem solving; exercises to improve strength, coordination, and balance; and information on evaluating home safety. The Roybal Center at Boston University developed the program, and the MaineHealth’s Partnership for Healthy Aging adapted MoB for volunteer lay leaders (13). The program has proven to be effective in reducing participants’ fear of falling, increasing confidence in managing fall risks, and increasing activity levels (14–16). MoB has an ROI of 144% for each dollar invested (17).
(4) *Assisted Home Safety Assessment*: the AHSA provides older adults at risk of falling home visits to identify and address environmental fall risk factors such as poor lighting, area rugs, or cords on the floor. Traditional Home Safety Evaluations are typically performed by a clinical practitioner, such as a visiting registered nurse (RN), PT, or occupational therapist (OT). When clinical personnel conduct home safety evaluations, they evaluate both the home environment and the individual’s physical ability to navigate the home based on their gait, strength, balance, and coordination. To test a lower cost alternative and to offer assessments to patients who were not eligible to receive a visit by an RN, PT, or OT due to insurance limitations, PWTF developed an innovative evidence-informed intervention to have this home visit performed by a CHW as an Assisted Home Safety Assessment (18–20). PWTF CHWs are trained to focus on the patient’s environment and they do not evaluate the patient’s functional status. When the CHW identifies a client who may have a fall risk based on a physical issue, s/he sends feedback to the referring clinic recommending further clinical assessment and/or services.

Prevention and Wellness Trust Fund developed standardized training and tools for the CHWs conducting AHSA. Subject matter experts consolidated various RN, PT, and OT home safety assessments (18–20) into a single screening tool appropriate for use by a trained CHW. Given the novelty of this intervention, the workflow and tools required multiple iterations to be developed and tested over the course of 2 years, between the 6-month planning and implementation phases of PWTF. Home safety evaluations conducted by the RN, PT, and OT have proven effective in reducing falls (1) and are included in the American Geriatrics Society/British Geriatrics Society Clinical Practice Guideline for the Prevention of Falls in Older Persons (9). However, research is still underway to evaluate the effectiveness of CHW home safety assessments on fall reduction.

### Program Implementation and Support

Funding of the nine PWTF partnerships began in early 2014 with a capacity building phase followed by an implementation phase beginning in January 2015.

Funding for the MDPH administration of the grantee program was legislatively capped at 15% of the total fund, approximately $8,550,000. Most of these funds were used to provide extensive training and technical assistance to support, coach, and educate partnerships. The team developed a statewide falls prevention learning collaborative adapted from the Institute for Healthcare Improvement Breakthrough Series framework for technical support, quality improvement (QI), and shared learning to accelerate improvement (21). Full-day statewide learning sessions are held twice per year and include plenary speakers focused on program topics, as well as, breakout sessions that facilitate site specific implementation sharing and networking opportunities. In addition to these bi-annual learning sessions, expert-led falls prevention webinars are offered several times per year.

The learning collaborative provides ongoing trainings on each intervention in multiple modalities. A contracted training organization provides instructor and coach trainings for Tai Chi and MoB. A subject matter expert delivered a multi-day and single-day AHSA training and subsequently developed an online AHSA training program for onboarding new staff. A STEADI content expert delivered on-site STEADI trainings for interdisciplinary teams that consisted of continuing medical education credits for physicians and continuing education unit credits for licensed nurses and allied health professionals. These provider and staff trainings have been conducted with teams at 14 clinical sites to date.

The learning collaborative also provides the structure and support for QI initiatives. PWTF staff developed a falls prevention program charter outlining the purpose of the collaborative and quantitative measures. Quarterly data-feedback reports track partnership progress on the falls-specific charter goals and provide an opportunity to assess areas of need and troubleshoot barriers to improvement. Using a data-driven QI model, teams are required to conduct Plan-Do-Study-Act (PDSA) cycles to test change concepts for improvement. Partnerships submit PDSA cycles quarterly to the MDPH PWTF team who in turn provide written feedback to the teams.

Technical support is provided to partnerships from a dedicated PWTF staff member focused exclusively on falls prevention who leads the collaborative with input from several subject matter experts who have expertise on the interventions. Toolkits and guidance documents have been developed in response to partnership challenges, needs and requests. To facilitate the sharing of resources, the PWTF team established a SharePoint webpage to provide a single repository for falls prevention related materials (research papers, clinical guidelines, PWTF guidance, training, tool kits, etc.). Each week an electronic “Weekly Update” newsletter is sent to partnerships to provide program updates, events, training opportunities, frequently asked questions, contract requirements, breaking news, and a myriad of other resources.

### Data Collection

Quarterly data collection from clinical (*n* = 23) and community-based organizations (*n* = 27) began in January 2015. The majority (55%) of the clinical sites submits data via the Massachusetts League of Community Health Centers through a software platform created by Azara Healthcare; on a nightly basis, EMR data are extracted and saved to a data warehouse. Other clinical sites either submit encounter-level EMR data extracts or an aggregate-level spreadsheet with the required data elements directly to MDPH. Community-based organizations are required to complete and submit a data collection spreadsheet for each of the interventions they implement that includes information on all clients for whom they receive a referral or who enrolls in the intervention. These data are compiled and analyzed by two MDPH epidemiologists and summarized in data reports provided to partnerships on a quarterly basis.

## Preliminary Findings

### Clinical STEADI Data

**Figure 3:**
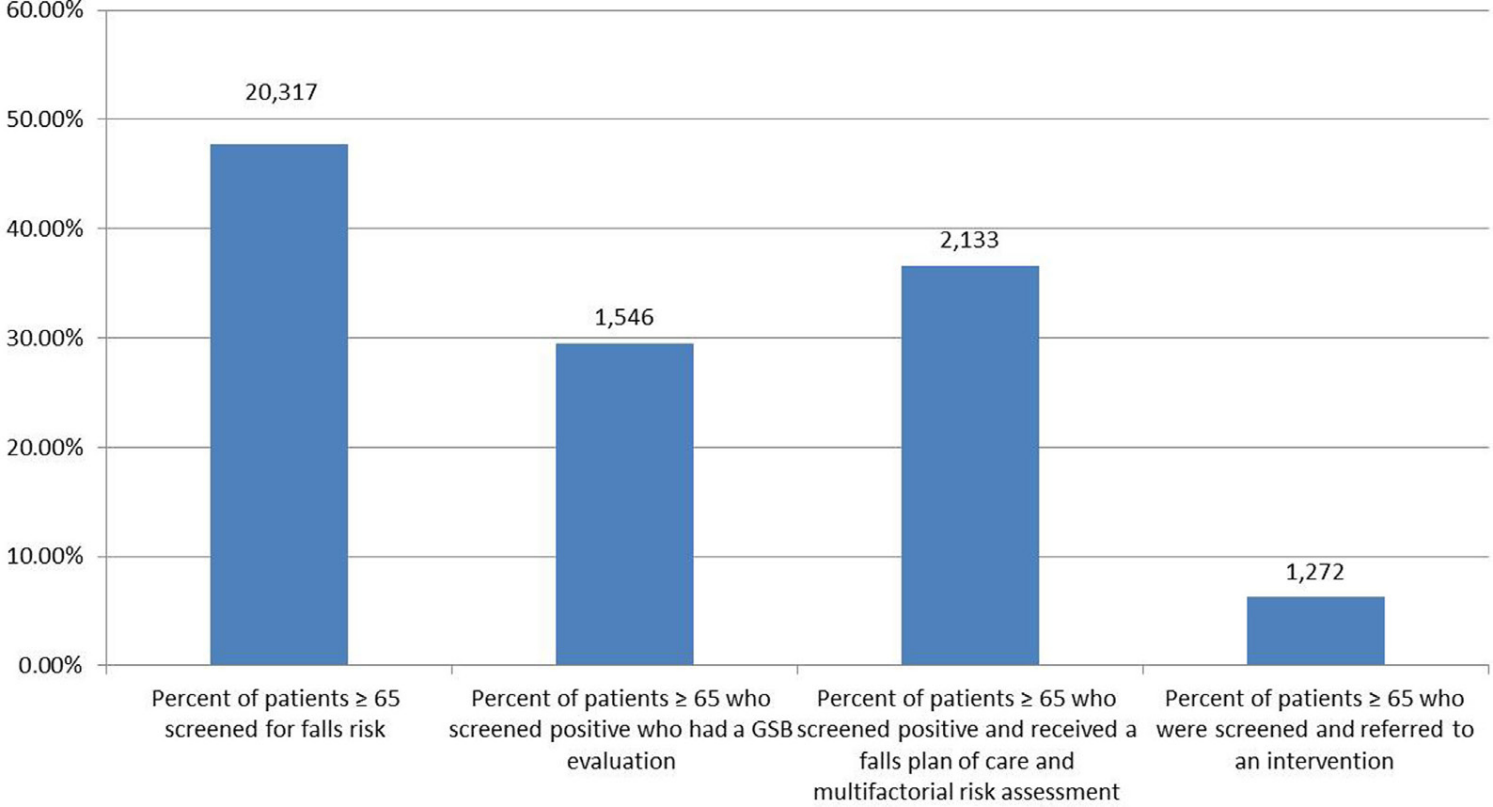
**PWTF clinical screening, assessment and referral data**. Bars have differing denominators based on patient eligibility.

As shown in Figure 3, the clinical sites implementing STEADI submitted data for the timeframe January to September 2016. During that 9-month period 48% (20,317) of patients aged 65 years and older were screened for falls risk and 30% (1,564) of those who screened positive received an evaluation of their gait, strength and balance [most often a Timed Up and Go (TUG) or “TUG” test] (22). Of those who screened positive, 37% (2,133) received a plan of care and a multifactorial clinical risk assessment. Of the patients screened, 6% (1,272) received referrals to a community falls prevention intervention (MoB, Tai Chi, or AHSA).

Stopping Elderly Accidents, Deaths and Injuries implementation was challenging for the PWTF primary care sites as falls risk assessment was a new area and it requires significant systems change. PWTF sites experienced many of the same implementation challenges as other sites nationwide (23), such as securing support from senior leadership and clinical staff; the lack of reimbursement for specific clinical components; no data fields in EMR to capture or assess falls assessments; and lack of workflows and processes for implementing STEADI.

### Community Program Data

**Figure 4:**
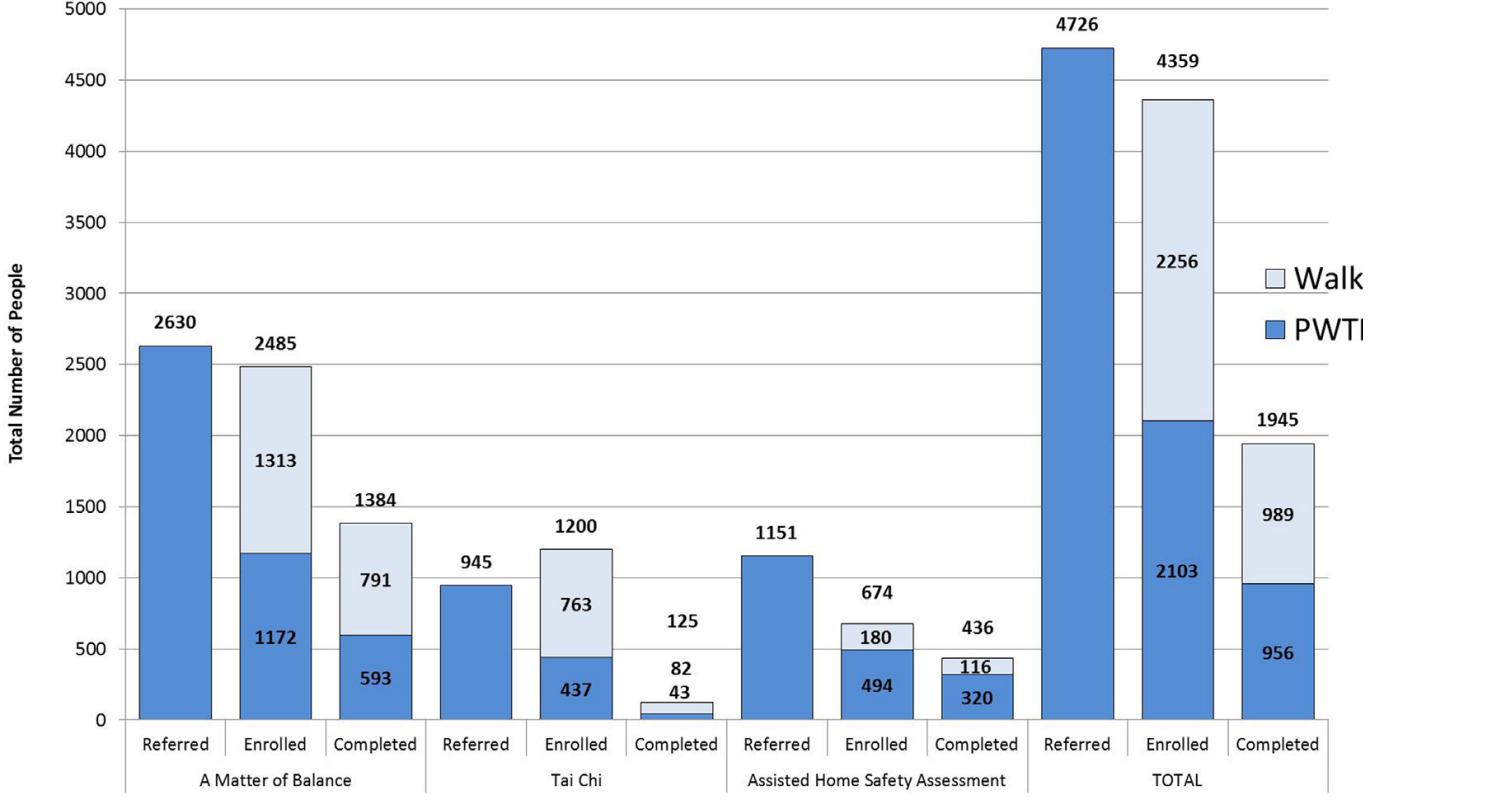
**PWTF fall prevention program referral, enrollment and completion data**.

Prevention and Wellness Trust Fund is currently in year two of the implementation phase of the program. Over a period of 21 months of implementation (January 2015 to September 2016), PWTF clinical sites assessed patients using the STEADI protocol and referred 4,726 individuals to PWTF community sites for falls prevention interventions. Of those referred, 44% (2,103) enrolled in the PWTF-sponsored community interventions and of those enrolled 45% (956) completed the interventions (refer to Figure 4). On average, for every five individuals referred, one individual completed the intervention. The clinical referrals have been increasing over time—the average falls referral rate in January 2015 was 141.5 referrals per month; by January 2016, the rate had increased to 225.3 referrals per month, representing a 60% increase in referral volume.

In addition to clinical referrals, PWTF community organizations are allowed to recruit individuals directly—without a clinical referral. These clients, referred to as “walk-ins” for PWTF purposes, are eligible for MoB and Tai Chi. Eligibility for AHSA requires risk screening and assessment of gait, strength and balance by staff at the community organization to determine risk level. During this same 21-month timeframe (January 2015 to September 2016), there were 2,256 “walk-ins” enrolled and 989 “completers.” Overall, with combined clinical referrals and “walk-ins,” more than 4,359 individuals enrolled, and approximately 1,945 completed interventions (refer to Figure 4).

Of the three falls interventions, MoB has received more referrals, enrollments, and completions than Tai Chi or AHSA. More than 50% of those enrolled in MoB or AHSA completed the interventions. Tai Chi currently shows much lower rates of completion (10%) because it is a significantly longer program (24 weeks) which creates a long time lag for Tai Chi completion rates (refer to Figure 4).

Clinical and community staff faced challenges in referring and enrolling individuals into community interventions due to barriers such as reluctance due to the time commitment, lack of understanding of risk, and unfamiliarity with programs or organizations running the programs. Partnerships tested multiple strategies to overcome these issues.

Analysis of the program’s impact on the prevalence of falls (with or without injuries), hospitalizations, and ED visits, as well as the impact of these prevention interventions on healthcare costs, will be part of a legislatively mandated independent program evaluation. That report will be released in 2017.

## Discussion

Massachusetts Department of Public Health PWTF staff and subject matter experts have provided support to PWTF clinic and community-based organization staff with all aspects of implementation such as training, troubleshooting challenges, sharing successful strategies, interpreting performance data, reviewing PDSA cycles, and developing tools. This section outlines key lessons learned during the implementation of falls prevention efforts across the eight partnerships.

1. Use patient-centered approaches, motivational interviewing techniques and face-to-face “warm hand-offs” for community program recruitment.
  Successful strategies for referring and enrolling individuals included:
  - Use of client-centered motivational interviewing techniques to communicate effectively with patients/clients about the value of the interventions and their readiness to enroll;
  - A joint letter or educational brochure from the clinical and community organizations describing the initiative and the name of the person who would be contacting them;
  - Primary care providers (physicians or mid-levels) promoting the interventions in addition to other staff who may be involved in the referral process (e.g., Medical Assistants, CHWs, referral coordinators);
  - “Warm hand-offs” or an in-person, face-to-face introduction from a clinical staff member to a community staff member for referrals. For example, a CHW from a clinical site making an in-person introduction of the patient to the CHW from the community site for the hand-off or referral. To further decrease barriers to enrolling in interventions, some of the community program staff schedules their office hours at the clinical sites to receive the face-to-face referrals.
2. Stopping Elderly Accidents, Deaths and Injuries implementation requires strong systems-level support.
  Implementing STEADI is a complex process that requires buy-in from senior leadership. Multidisciplinary resources are needed, including administrative leaders to commit the required resources; QI staff to create workflows and design tests of change; IT staff to support EMR changes and data collection; clinical champions to train and support teams to develop and test QI efforts; and front-line staff to conduct screenings and assessments. Many PWTF clinical sites lacked these supports, which negatively impacted or delayed their STEADI implementation efforts. Sites did not fully understand the breadth of requirements and MDPH provided limited guidance or training on STEADI implementation until January 2016. The training for STEADI is now in place and has helped to clarify expectations for clinical staff. Teams are also better prepared for the systems-level changes needed to be successful.
3. Use team-based approaches to implement the STEADI multifactorial risk assessment.
  Patients who are at high-risk of falls require a comprehensive clinical assessment to assess and treat their clinical risk factors (e.g., foot exam, medication review, vision check, etc.). The comprehensive assessment is time consuming and difficult to complete during a routine office visit, especially when there is a competing clinical priority. To address this challenge, sites developed various strategies. One approach was to address elements of the assessment over several visits instead of trying to complete all of it during one visit. Another strategy was implementing a falls clinic—a visit dedicated to completing the multifactorial falls assessments on high-risk patients. Sites have also identified specific components of the assessment that can be performed by other clinical team members to reduce the burden on the primary provider. These strategies allow staff to work at the top of their licenses, take advantage of new learning opportunities and build the skills needed to become falls prevention champions.
4. Begin work on STEADI EMR templates early.
  Clinical guidelines for falls prevention have not been widely adopted in routine clinical care and most EMRs do not include templates for falls screening and assessment. Therefore, few of the PWTF clinical sites were initially able to collect structured data on their falls prevention work. Custom additions to EMRs are costly and may require significant wait times. In addition, deciding which and how many fields to incorporate into EMRs is complicated by differences between national quality metrics (e.g., NQF 0101) and toolkits such as STEADI. This delayed the creation of electronic alerts, automated data collection, and real-time reporting that make practice change consistent and sustainable. Clinical sites should develop STEADI EMR templates prior to practice-wide STEADI implementation.
5. Collect health outcome and quality measures in addition to quantity measures.
  Prevention and Wellness Trust Fund focused on collecting data on referral, enrollment, and completion metrics for the community falls work. An ideal measurement strategy would also include measures of the quality of programs (number of home modifications, contacts/calls with CHWs, or class evaluations) and interim health outcome measures (reduced fear of falling, increased mobility, or improved strength). If possible, organizations should create partnerships with local hospitals to regularly obtain data on hospitalizations and ED visits for fall-related injuries so the impact of the interventions could be assessed and targeted toward populations most in need.
6. Promote Assisted Home Safety Assessment as an approach to keep individuals independent and safe in their homes.
  Some older adults are reluctant to allow a CHW into their home because they fear they may be forced to leave their home as a result of a negative assessment. Fortunately, these challenges can be mitigated when clinicians and staff educate the patient and use behavioral change tactics such as brief motivational interviewing. When patients understand that the home assessment is a means to keep them safe in their home and to provide them with helpful resources, clients are more apt to accept the AHSA. As an additional step to encourage enrollment mentioned above some of the PWTF teams have tested mailing a joint letter from the clinic and the community partner to the referred patients emphasizing the goal of the assessment.
7. Allow patients at moderate- and high-risk for falls to be eligible for Assisted Home Safety Assessments.
  Initially, assisted home safety assessment eligibility was limited to patients who had been screened by their PCP and determined to be at high risk for falling, as per the STEADI algorithm. This limited initial referrals. In addition, the clinical sites were not ready to refer immediately as they needed time to establish or improve their screening and referral services during the first year of implementation, leaving the staff trained to conduct home assessments underutilized.
  To address this, MDPH relaxed the restrictions in two ways. First, MDPH allowed both high- and moderate-risk patients eligibility for AHSA. This provided an opportunity for existing CHW teams to reach a wider population and increase prevention opportunities. Second, PWTF community sites were permitted to screen and conduct the TUG test and enroll those at moderate- or high-risk for AHSA (22). Realizing this was breaking away from the STEADI algorithm, which starts at the clinical site; community sites were required to refer high-risk clients back to their PCP for a clinical assessment. While this created other challenges (e.g., training, data collection, workflow, etc.) both of these changes successfully improved falls outreach to patients in the participating communities.
8. Coordinate community-based CHWs and Home Health Agency staff to optimize the impact of services provided.
  High-risk patients with full Medicare may be eligible for an OT/PT/VNA home safety evaluation, but providers may not refer to that service due to lack of awareness of the benefit. Provider education is needed so that eligible patients are referred for home safety evaluations as appropriate.
  For some individuals, both the OT/PT/VNA home visit and the CHW home visit are valuable. Individuals participating in the CHW home visit may need an OT/PT/VNA assessment to determine the need for grab bars and other equipment. After an OT/PT/VNA assessment, a CHW may be needed to follow up on outstanding issues. Ideally, the organizations and staff involved in conducting these two types of home visits coordinate their services and share information. The methods of coordination vary between clinical sites, but those relationships are essential to improve efficiency, minimize duplication, and improve the patient’s experience. Several PWTF partnerships have begun working with their Home Health Agencies to achieve these goals.
9. Assist with purchase and installation of durable medical equipment for falls prevention that is not covered by Medicare.
  Several items, including, but not limited to raised toilet seats, grab bars, stair railings, and shower chairs, are often recommended by PT/OT following a home evaluation. However, Medicare often does not cover these items or installation. PWTF offered a home modification budget of $500 per person receiving an AHSA to purchase and install necessary items for patients/clients who could not otherwise afford needed equipment. Depending on the region, CHWs were sometimes able to get equipment from the Area Agency on Aging or a local non-profit at a discounted price. The AHSA protocol requires CHWs/clinics to explore other funding sources before using these PWTF funds.
10. Enroll twice as many participants as needed to meet MoB class completion goals.
  Matter of Balance and Tai Chi are group classes that are more cost-effective and beneficial when meeting recommended enrollment numbers. Some programs’ fidelity requirements specify a minimum number of enrollees to hold the class. In the initial program roll-out, community sites experienced significant no-shows for the first class, as well as, high levels of drop-outs over the course of the program. When possible, program staff followed up to identify attendance barriers and tested strategies to address common challenges, such as lack of transportation, language barriers, misunderstanding about the program and its risks, and location or timing of class. Based on PWTF enrollment and completion data from 21 months of MoB implementation, on average, 50% of enrollees completed. Therefore, when planning, sites need to consider enrolling twice as many people to meet their class attendee goals. Tai Chi completion rates are even lower due to the long duration of the program, therefore, enrollment goals should be more than double the number expected to complete.
11. Allocate funds for transportation to assist older adults in attending community falls prevention programs.
  Many older adults lacked transportation options for attending classes. This barrier created recruitment and retention challenges, especially for those participants with the fewest resources and greatest need for these programs. CHWs connected older adults to local transportation resources. When this was not possible, several organizations used PWTF funds for transportation services, such as a taxi voucher system. Removing this barrier led to improvements in attendance. The long-term goal would be to have more locally accessible classes to reduce the need or decrease the cost for transportation to and from home.
12. Use bilingual trained patient champions to serve as MoB coaches to address language barriers.
  Prevention and Wellness Trust Fund partnerships include areas with linguistically diverse populations. MoB and many of the other PWTF community-based interventions for hypertension and diabetes have been adapted for Spanish speakers; however, there is an outstanding need for many more languages. One strategy to address this is to recruit and train bilingual patient champions as coaches.
13. Offer DVDs and allow participants to log home practice time to improve Tai Chi completion rates.
  Tai Chi must be practiced for a total of 50 h before benefits are seen (24). However, recruiting participants for such a significant time commitment proved difficult. Successful strategies to address this were offering DVDs and allowing participants to log home practice time; providing graded incentives after specific time intervals; breaking the total program into multiple sessions; and encouraging participants to try the program without focusing on the duration.

## Conclusion

Several prevention interventions are proven to be effective at reducing falls and falls risk. However, coordinated and comprehensive approaches to falls prevention are not currently widespread. The PWTF has provided the state an opportunity to test a new model for integrated, multifaceted falls prevention efforts that span and link clinical and community interventions. As a result of this project, PWTF is uncovering and capturing important lessons learned to guide future programs and models.

Sustainable reimbursement mechanisms are needed to integrate falls prevention systematically. Reimbursement mechanisms for clinical falls prevention work are not straightforward, and this presents challenges for buy-in and sustainability. EMR templates to collect STEADI data elements must be required for falls assessment to be tracked and assessed. The sustainability of community interventions (MoB, Tai Chi, and AHSA) depends on funding or adoption by ACOs. Finally, in order to reach the most vulnerable and at-risk patients, programs must address language gaps and provide resources for transportation.

Creating strong clinical and community partnerships are essential for provider participation, successful referrals and client participation. If clinical providers are confident about the availability, effectiveness, and quality of community programs to refer their at-risk patients they will be motivated to screen and refer patients. Furthermore, successful patient referrals happen as a result of effective linkages with people-centered approaches that involve strong relationships; face-to-face interaction; and brief motivational interviewing techniques that assess readiness and provide supportive coaching. The learning collaborative model has been effective for bringing teams across the state together for shared learning and is an essential component for building and enhancing successful clinical and community partnerships.

A strong foundation has been built as a result of this innovative approach to falls prevention. The progress achieved and lessons learned can be used to inform future programs. As our population ages, people continue to live longer and the incidence of falls increases, the implementation of evidence-based prevention programs that reduce healthcare costs will be an urgent imperative for policy makers, payers, providers, and older adults.

## Author Contributions

LC: provides leadership program design and implementation; contributed to the paper outline and drafted sections; reviewed and contributed to multiple versions; incorporated edits from authors and reviewers; approved final version; and coordinated review and submission process. JJ: supports program implementation; participated in developing the paper outline; drafted sections; reviewed and contributed to multiple versions; incorporated edits from authors and reviewers; and approved final version. SH: supports program implementation; developed initial outline; convened authors; drafted sections; created tables and figures; reviewed and contributed to multiple versions; incorporated edits from authors and reviewers; and approved final version. KS: supports program implementation; participated in developing the outline; drafted sections; reviewed and contributed to multiple versions of text, tables, and figures; and approved final version. PM: supports program implementation; participated in developing the outline; drafted sections; reviewed and contributed to multiple versions; and approved final version. AB: provides leadership on program data collection and analysis; conducted data analysis and interpretation; and approved final version. JZ: provides leadership on program implementation; participated in developing the outline; reviewed and contributed to multiple versions; incorporated edits from authors and reviewers; and approved final version.

## Conflict of Interest Statement

The authors declare that the research was conducted in the absence of any commercial or financial relationships that could be construed as a potential conflict of interest.
